# Encapsulation of lutein in nanoemulsions: Comparative evaluation of chickpea and soy protein isolates on physicochemical stability, antioxidant activity, and rheological properties

**DOI:** 10.1016/j.fochx.2025.102623

**Published:** 2025-06-03

**Authors:** Farhang Hameed Awlqadr, Babak Ghanbarzadeh, Ammar B. Altemimi, Khaled Arab, Saeed Dadashi, Akram Pezeshki, Mohammad Ali Hesarinejad, Tarek Gamal Abedelmaksoud

**Affiliations:** a Food Science and Quality Control, Halabja Technical College, Sulaimani Polytechnic University, Sulaymaniyah, Iraq; b Department of Food Science and Technology, Faculty of Agriculture, University of Tabriz, Iran; c Department of Food Science, College of Agriculture, University of Basrah, Basrah 61004, Iraq; d Department of Food Sensory and Cognitive Science, Research Institute of Food Science and Technology (RIFST), Mashhad, Iran.; e Food Science Department, Faculty of Agriculture, Cairo University, Giza 12613, Egypt

**Keywords:** Lutein nanoemulsion, Chickpea protein isolate (CPI), Soy protein isolate (SPI), Encapsulation efficiency, Rheological stability, Bioavailability

## Abstract

Nanoemulsions have emerged as advanced systems for encapsulating bioactive compounds, offering enhanced stability, bioavailability, and functionality in various applications. This study evaluates the potential of chickpea protein isolate (CPI) and soy protein isolate (SPI) as emulsifiers in the preparation and stabilization of lutein-loaded nanoemulsions. The study looked at CPI and SPI and how each of them interacted with the physical and chemical properties, antioxidant activity, and stability of proteins at different concentration levels (1 %, 3 %, and 5 %). The data showed that CPI was better than SPI in terms of equal sizing, zeta potential, and long-term stability. Nanoemulsions made using 3 % CPI gave the best results showing optimal particle size, antioxidant retention, and rheological stability. CPI-stabilized emulsions, which were the result of strong thixotropic behavior higher hysteresis loop areas and more robust hydrogen bonding and cohesive interfacial layer, were the better products. In contrast, SPI-stabilized emulsions were less efficient because of their reliance on hydrophobic interactions. In addition, lutein encapsulation was a mean to increase the stability of emulsions and also to boost up the antioxidant efficiency against blank formulations. The results show the excellent emulsifying capabilities of CPI and the application of bioactive ingredients in functional foods and nutraceuticals that benefit the bioavailability and function of lutein. The findings can pave the way for the utilization of plant-based proteins for eco-friendly nanoemulsion technologies to be used in bioactive delivery.

## Highlights

• Chickpea protein isolates (CPI) excelled over soy in lutein nanoemulsion stability.
• Nanoemulsions with 3 % CPI showed optimal particle size and antioxidant retention.
• CPI demonstrated strong hydrogen bonding for lutein encapsulation, enhancing stability.
• Higher protein concentrations improved emulsification and reduced droplet aggregation.
• Findings support CPI's role in eco-friendly, bioactive delivery in food products.

## Graphical abstract

**Figure:**
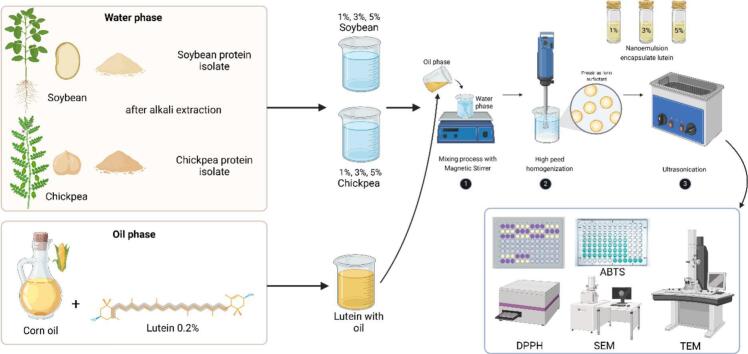

## 1 Introduction

Nanoemulsions have emerged as a versatile and innovative tool in food science, pharmaceuticals, and cosmetics, offering significant advantages over traditional emulsion systems. Identifying as colloidal systems characteristic with droplet sizes measuring 20 to 200 nm and nanoemulsions show remarkable physical stability increased bioavailability and higher optical clearness as compared to conventional emulsions (Jacob et al., 2024). The key properties attributed to small droplet size, which result in minimal gravitational separation and droplet aggregation, making nanoemulsions the best choice for encapsulating and delivering hydrophobic bioactive compounds in functional foods and beverages. Nanoemulsion delivery systems better suit lutein, a well-known carotenoid as compared to any other medicine. These compounds are acquired through feeding, then after being incorporated into foods, they convey benefits such as better health and reduce the risk of disease. Out of the pool of nutrients that are found in high concentrations in leafy green vegetables, marigolds, and other plant sources, lutein is one of them and it is well-known for its strong antioxidant properties. Plus, lutein plays a most important role in the eye's defense system by enhancing vision and scavenging those bad rays that come from the sun. It thus gains a protective function against age-related macular degeneration (AMD) (Manzoor et al., 2023). Lutein's usefulness as a viable property in food systems is actually limited by its poor solubility in water and its easy susceptibility to oxidation. The technologies of encapsulating it in the nanoemulsions give a real possibility to cope with the above-mentioned problems by optimizing the stability or the bioavailability of lutein which is achieved (Du et al., 2023). This is a research that is about how articulation proteins make nanoemulsion systems effective by their amphiphilic characteristic, which in turn allows them to be able to stabilize the interface of oil and water. Chickpea protein isolates (CPI) and soy protein isolates (SPI) are common types of plant-based proteins. Chickpea protein isolates, obtained from chickpeas, contain good emulsification properties, eliminate allergic risks such as hypersensitivity symptoms, and deliver a high nutritional value. All the description of labeled chickpea proteins, in particular, had some stability revelation, like in acidic food systems, and corresponded to their globular proteins composition such as vicilin and legumin being as well as the robust interfacial films (Xinyu Zhang et al., 2023). SPI, which is the protein of concern in soybean, is widely investigated because it has a great amount of both β-conglycinin and glycinin which are excellently functioning as emulsifiers. The state of these proteins and the structure they have allow them to accomplish the required tasks. The small sizes of the droplets are enabled to be stabilized through the silicon polymers injection process (Pereira, Jun, Li, Wall, & Ho, 2023). Lutein encapsulation in CPI- as well as SPI-stabilized nanoemulsions is an excellent solution for several crucial issues. To begin with, the remarkably small droplet size, which is a standout feature of nanoemulsions, makes the bioavailability of lutein much better by providing more area to the surface and so encouraging quick digestion and absorption in the intestines (R. Wang et al., 2023).For instance, (Toragall et al., 2021) developed an oleic-linoleic acid nanoemulsion that improved lutein's solubility by over 700-fold and significantly boosted its bioavailability in vivo. (Liu et al., 2021) compared different lipid-based carriers and found nanoemulsions stabilized with zein peptides increased lutein bioavailability up to 62 %. In addition (Teo et al., 2017) reported higher cellular uptake of lutein in Caco-2 cells using whey protein-stabilized nanoemulsions compared to conventional emulsions. Murillo et al. (2016) showed that a lutein nanoemulsion doubled plasma and liver lutein levels in guinea pigs compared to powdered lutein, confirming improved bioavailability (Murillo et al., 2016). Secondly, the oil droplets sheath over lutein, as well as the food coatings made by using CPI and SPI, making it difficult for the light, heat, and oxygen to affect the lutein's chemical structure and thus making it more stable (Y. Cheng et al., 2025). Nanoemulsion technology has some properties that are pretty amazing, such as being able to transport lutein through functional foods and nutraceutical products. The most common procedure for the preparation of nanoemulsions involves methods such as high-pressure homogenization, and ultrasonication, and then ultrasonical or high-pressure homogenization are commonly used for the preparations of the nanoparticles. This is the standard technique by which the ultra-fine oil droplets are created of shear forces which is later stabilized by using the emulsifiers like CPI and SPI. The choice of emulsifier is very crucial because it has several effects on the stability, particle size, and bioavailability of nanoemulsions. Those proteins with balanced hydrophilic-hydrophobic regions and the high molecular flexibility are the ones that are found in CPI and SPI and are very effective in stabilizing the nanoemulsions (Yang et al., 2022).

CPI and SPI are two plant-based ingredients with different functional features that are often used in different applications. The capacity of CPI to emulsify effectively at low pH is especially beneficial for such products as fruit-based beverages and other low-pH foods. The plenteous solubility and surface activity of CPI are also what makes it develop stable nanoemulsions even at very low concentrations (Hei et al., 2024). Lutein stability and bioactiveness are advanced by incorporating nanoemulsions that are CPI- and SPI-stabilized into functional foods for the manufacturers to provide innovative products that meet the dual challenges of health and sustainability. Technology of nanoemulsions that are made by lutein bioavailability improvement also makes actual controlled release, which guarantees a constant supply of the bioactive compound in the organism. As is generally the case, prolonged administration is necessary for the lubrical of such diseases as AMD. This is simply a case in point, long-term supplementation is actually the only way to achieve therapeutic effects (Nunes, Hashemi, Beyer Gregersen, et al., 2023). Besides that, the possibility of focusing more on lutein-containing nanoemulsions in everyday food products like drinks, dairy items, and confectionery definitely makes it a convenient and more pleasant way to get in the beneficial compound to the people diet.

The purpose of this research is to investigate the emulsifying capacity of CPI and SPI in the creation and protection of lutein-loaded nanoemulsions. This study reveals that besides getting the best nanoemulsions through studying the basic properties of these nanoemulsions, the formulation will be chosen which will improve lutein's bioavailability and stabilization. The findings from the study will be an addition to the available information on plant-based proteins in nanoemulsion systems and give insights into the formation of functional foods that improve the immune system by means of bioactive materials. This work, in a nutshell, illuminates the possible solutions to solving the global health challenges while being sustainable in the Maldivian food systems through the use of creative encapsulation technologies.

## 2 Materials and methods

### 2.1 Materials

Chickpea (*Pisum sativum L*.) and soybean (*Glycine* max) seeds were sourced locally in Tabriz, Iran, and used to extract (CPI) and (SPI). All reagents used in this study were of analytical grade and included lutein, n-hexane, ethanol, DPPH, ABTS, and other standard chemicals, which were purchased from Merck (Germany). For instrumental analysis, used for the study included a high-shear homogenizer (HEIDOLPH, Germany), an ultrasonic probe (Sonics & Materials, USA), a centrifuge (Fan Azma Gostar, Iran), a spectrophotometer (Spectrum Filler Scientific, China), and a Nano Zetasizer (Malvern Instruments Ltd., UK) Additional tools included a Fourier-transform infrared spectrometer (FTIR, Bruker, Germany) and microscopy instruments (TEM and SEM, Zeiss, Germany, and TESCAN, Czech Republic).

### 2.2 Preparation and characterization CPI, SPI, and nanoemulsions loaded lutein

#### 2.2.1 Preparation of (CPI) and (SPI)

Chickpea and soybean protein isolates were prepared using alkaline extraction and isoelectric precipitation. Seeds were cleaned, ground into fine flour, and defatted with n-hexane (1:5 *w*/*v*) under stirring for 2 h. The defatted flour was dried and suspended in distilled water (1:10 w/v), and the pH was adjusted to 10 with 1 N NaOH to solubilize the proteins. After 2 h of stirring, the mixture was centrifuged at 4000 rpm for 30 min. Protein-rich supernatants were adjusted to pH 4.5 by adding 1 N HCl in order to cause isoelectric precipitation. Precipitated proteins were centrifuged, washed with distilled water, neutralized, freeze-dried, and stored for further use. This technique employs methods that are well-established in the protein isolation of plants (Shrestha et al., 2023).

#### 2.2.2 Determining the solubility of CPI and SPI

(CPI) and (SPI) were analyzed for solubility over the entire pH range (3 to 11). For the study, 1 % w/v of protein solutions were made by the solubilization of the isolates in distilled water. The pH values were adjusted by the sequential use of 1 N NaOH or 1 N HCl. The supernatant after centrifugation of the samples at 10,000 rpm for 10 min was analyzed for protein quantification following the Bradford method.

To calculate solubility (%), we implemented the given formula.

Solubility (%) = (Total protein in sample protein in supernatant) × 100

This method is in tandem with the norms already set up for the analysis of the protein solubility at varied pH levels (Khushairay et al., 2023)**.**

#### 2.2.3 Preparation Nanoemulsions loaded with lutein

Nanoemulsions that carried lutein were produced by high-energy emulsification methods. Lutein was first dissolved in corn oil (5 %) at a concentration of 0.2 % (*w*/*v*) under continuous stirring at room temperature for 1 h in a dark chamber for preventing its degradation. For the aqueous phase, (CPI) or (SPI) was dissolved in distilled water at concentrations of 1 %, 3 %, and 5 % (w/v). The oil phase with lutein was slowly poured into the water phase and then pre-emulsified with the help of a high-shear homogenizer (15,000 rpm) for 15 min. The obtained coarse emulsions were treated further with an ultrasonic probe (20 kHz) for 10 min to reach nanoemulsion droplet sizes. One-minute intervals were given between the ultrasound cycles to avoid overheating. The plant-based nanoemulsions that were produced were then stored in dark containers at room temperature for the upcoming analysis. The method of stabilization through protein isolates for the creation of nanoemulsions transporting lipophilic bioactives is in line with previous studies (Y. Wang, M. Geng, et al., 2023b).

### 2.3 Investigating the physicochemical properties of nanoemulsions

#### 2.3.1 Average particle size and particle size distribution

Particle size distribution and *Z*-average were taken as the average particle size of the nanoemulsions prepared using a method of dynamic light scattering (DLS) with a Nano Zetasizer (Malvern Instruments Ltd., UK). Before the measurement was performed, the samples were diluted with distilled water (1:50 *v*/v) to reduce multiple scattering effects. Measurements at 25 °C were taken and the polydispersity index (PDI) was calculated to evaluate droplet size distribution uniformity. Readings on the 1st, 7th, and 15th days of storage were taken to assess the nanoemulsions' stability over time (Y. Wang, M. Geng, et al., 2023a).

#### 2.3.2 Zeta potential of particles

To determine the zeta potential of nanoemulsions, which gives an indication of the surface charge of the droplets, the same Nano Zetasizer was used and subjected to electrophoretic light scattering (ELS) mode. At 25 °C measurements were conducted with undiluted samples. Zeta potential values were employed to evaluate the electrostatic stability of the emulsions, whereby higher absolute values represented greater cohesion in comparison with the results after reporting the data (Glikman et al., 2024).

#### 2.3.3 Fourier-transform infrared spectroscopy (FTIR)

Fourier-transform infrared spectroscopy (FTIR) was employed to examine the protein isolates – lutein interactions in the nanoemulsions. Samples were freeze-dried and ground into fine powders and IR was used to scan them in the range of 4000–400 cm^−1^ which was carried out by an FTIR spectrometer (Bruker, Germany). Peaks corresponding to the various functional groups were determined so as to detect the presence of hydrogen bonding or electrostatic interactions between the emulsifier and lutein. This analysis was the marble that provided deep knowledge about the encapsulation mechanism and the structural integrity of the emulsions (Y. Cheng et al., 2025).

### 2.4 Investigating the antioxidant properties of Nanoemulsions

#### 2.4.1 DPPH test

The antioxidant activity of nanoemulsions was thoroughly assessed by carrying out the chemical experiment known as the DPPH (2,2-diphenyl-1-picrylhydrazyl) free radical scavenging assay. A solution of 0.1 mM DPPH in methanol was prepared. In order to do the experiment, 1 mL from each sample of nanoemulsions was added to 3 mL of the DPPH solution and in the meantime incubated in the dark at room temperature for 30 min. At 517 nm, the absorbance of the prepared mixture was determined with the help of a UV–Vis spectrophotometer. The antioxidant activity (%) level was computed using the next formula:

Antioxidant Activity (%) = [(A₀ − A₁)/A₀] × 100

where A_0_ is the absorbance of the control (DPPH without sample) and A_1_ is the absorbance of the sample. Higher percentages show more effective antioxidant activity (Kumar, Shikha, & Sinha, 2024).

#### 2.4.2 ABTS test

Further investigation of the antioxidant action of nanoemulsions was done with the help of the ABTS (2,2′-azino-bis(3-ethylbenzothiazoline-6-sulfonic acid)) radical cation decolorization assay. Stock solution of ABTS was prepared by mixing 7 mM ABTS with 2.45 mM potassium persulfate and allowing the mixture to react in the dark at room temperature for 12–16 h to yield ABTS^+^ radicals. The working solution was made by dilution of the stock solution with phosphate-buffered saline (PBS) to obtain an absorbance of 0.7 ± 0.02 at 734 nm.

During the experiment, 20 μL of the nanoemulsion sample and 2 mL of the ABTS working solution were thoroughly shaken together, after which, they were placed in the dark for 6 min. The absorbance was measured at 734 nm, and the antioxidant activity was calculated using a Trolox standard curve. Results were expressed as Trolox Equivalent Antioxidant Capacity (TEAC, μM) (de Farias et al., 2024).

### 2.5 Investigating the morphology of nanoemulsion systems

#### 2.5.1 Transmission electron microscope (TEM)

A transmission electron microscope (TEM, LEO 906, Zeiss, Germany) images nanoemulsion droplets' size and shape. Sample preparation involved placing 8 μL of each nanoemulsion on a carbon-coated copper grid and staining it with 2 % phosphotungstic acid. The sample was air-dried after filter paper removed excess stain. Image of droplets taken at 100 kV acceleration. TEM investigation revealed nanoemulsion droplet size, shape, and homogeneity (Sithole et al., 2021). Micrographs were taken at a magnification of 30,000× to clearly visualize the structure of the emulsified particles.

#### 2.5.2 Scanning electron microscope (SEM)

A scanning electron microscope (SEM, MIRA3, TESCAN, Czech Republic) studied nanoemulsion droplet surface morphology. Spread samples equally on a flat surface and dry at 37 °C. A sputter coater coated the dry samples with a thin gold layer on aluminum stubs. SEM photography under high vacuum captured emulsion droplet surface features and possible aggregation (Zeng, Lee, Jo, & Choi, 2023). SEM images were captured at a magnification of 25,000× to observe surface features in detail.

### 2.6 Efficacy of encapsulation and investigation of chemical stability of encapsulated lutein

The nanoemulsions' lutein encapsulation effectiveness (EE%) was measured spectrophotometrically. Mixing a 200 μL nanoemulsion sample with 1 mL ethanol disrupts the emulsion and releases lutein. After adding 2 mL of hexane, the liquid was vortexed for 1 min to extract lutein. The solution was centrifuged at 8000 rpm for 20 min to separate phases. A UV–Vis spectrophotometer evaluated hexane layer absorption at 445 nm. Lutein concentration was estimated using a standard curve. The formula for encapsulation efficiency was:

EE% = Total Added Lutein (mg)/Encapsulated Lutein (mg) × 100

The chemical stability of encapsulated lutein was evaluated during storage at refrigerator temperature (4 °C) for 15 days. Lutein stability (ES%) was calculated as:

ES% = Encapsulated Lutein at Day 0 (mg)/Lutein Remaining (mg) × 100

This approach revealed lutein retention in emulsion droplets and storage stability. These assessments follow bioactive chemical nanoencapsulation research methods (Li et al., 2023).

### 2.7 Creaming stability of nanoemulsions

The creaming index (CI%) was measured by the physical stability of the nanoemulsions over 14 days of storage at room temperature. Nanoemulsions were stored in fleeting graduated tubes and left to stand undisturbed. Cream layer's (Hc) height, the height of the liquid layer (Hs), and the total height of the emulsion (Ht) were measured on days 1, 7, and 14. The creaming index was obtained using the formula:

CI% = (Hc + Hs)/ Ht  × 100

Lower CI% implies the better physical stability of the emulsion, as it indicates the reduction of gravitational and phase separation. This technique has a lot of uses in detecting the stability of emulsions be it in the food or pharmaceutical industry (Paulo et al., 2023).

### 2.8 Investigating the rheological properties of nanoemulsions

The rheological properties of the nanoemulsions were assessed by a rheometer (Anton Paar Smartpave 102, Austria) configured with parallel plate geometry (50 mm diameter, 0.206 mm gap size). Sodium azide (0.02 % *w*/*v*) was mixed into every sample to hinder the growth of microorganisms throughout the analysis process.

#### 2.8.1 Shear stress–shear rate relationship

The flow behavior and apparent viscosity of the nanoemulsions were measured by applying a shear rate against the temperature of 25 °C while varying the shear rate from 0.01 s^−1^ to 100 s^−1^. The procedure was used by this study to identify whether the emulsions had Newtonian behavior or a non-Newtonian behavior (shear-thinning or shear-thickening). At 16 different points, data were collected and plotted as shear stress versus shear rate to have a clear flow curve (Prakash & Parida, 2023).

#### 2.8.2 Thixotropic properties

Thixotropic behavior was assessed using a hysteresis loop test. The test comprised three stages:

1. Increasing the shear rate from 0.01 s^−1^ to 100 s^−1^ over 160 s.
2. Holding at 100 s^−1^ for 5 s.
3. Reducing the shear rate back to 0.01 s^−1^ over 160 s.

The area between the ascending and descending flow curves (hysteresis loop) was calculated to quantify thixotropic behavior. Larger hysteresis areas indicate greater structural recovery delays upon the cessation of shear (Pincot et al., 2023).

### 2.9 Statistical analysis

A completely randomized design (CRD) was employed to evaluate the effects of protein type (chickpea or soy protein isolate) and concentration (1 %, 3 %, and 5 % *w*/*v*) on the properties of lutein-loaded nanoemulsions. Data, including particle size, zeta potential, antioxidant activity, and encapsulation efficiency, were analyzed using ANOVA at a 95 % confidence level (*p* < 0.05). Tukey's HSD test was applied for post hoc comparisons. Results were expressed as mean ± standard deviation (SD) and analyzed using SPSS software (version 26.0).

## 3 Results and discussion

### 3.1 Preparation and characterization CPI, SPI, and nanoemulsions loaded lutein

#### 3.1.1 Determining the solubility of CPI and SPI

**Fig. 1:**
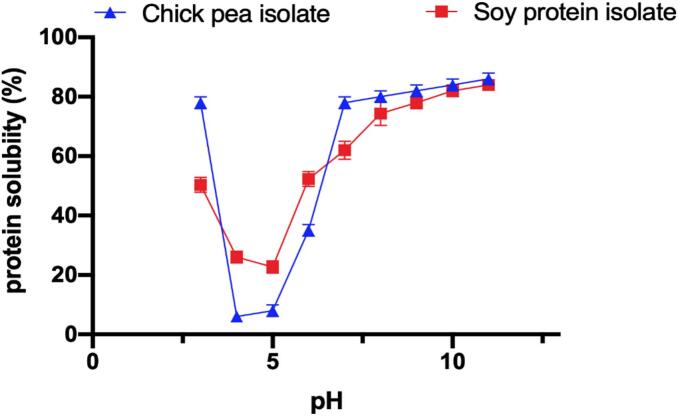
Protein solubility (%) of (CPI) and (SPI) for the whole pH range from 3 to 11 shows pH values: pH 4–5 solubility levels are very low near the point of maximum solubility while the conditions of high acidity (pH < 3) or high-alkaline reactions (pH > 7) prove the opposite.

The solubility profiles of (CPI) and (SPI) across the pH range of 3 to 11 are presented in Fig. 1. The results revealed that both CPI and SPI exhibited pH-dependent solubility, with minimum solubility observed near their isoelectric points (pH 4.0–5.0) and maximum solubility at more acidic (pH < 3.0) or alkaline (pH > 7.0) conditions. CPI demonstrated a higher aqueous solubility at lower pH compared to SPI. This was probably the effect resulting from the particular structure and amino acid composition of the proteins (Xin Zhang, Fan, Liu, & Li, 2023). The CPI's high water-solubility in acidic circumstances could make it fit for several applications in low pH food systems such as fruit-based beverages. Indeed, on the other hand, the solubility of SPI at elevated pH was illustrated qualitatively to be more during the alkaline conditions, which was due to the strong interactions of glycinin and β-conglycinin fractions, those were more stable at higher pH levels (M. Zeng, Lee, Jo, & Choi, 2023). These data will be useful for the preparation of lutein-nanoemulsions since protein solubility is the direct factor of emulsion stability and droplet size. CPI or SPI can be the choices for the production of nanoemulsions based on the pH range of the final product.

### 3.2 The effect of surfactant type and concentration on particle size, PDI, and zeta potential of lutein-loaded Nanoemulsions

The research team tested the efficiency of (CPI) and (SPI) in creating lutein-loaded nanoemulsions, which confines all the size, polydispersity index (PDI), and zeta potential measurements made within 15 days. The results, summarized in Table 1, reveal significant differences in the stability and physical characteristics of the nanoemulsions based on surfactant type, concentration, and storage time. For the initial particle size of nanoemulsions varied significantly based on the type and concentration of the emulsifier. At Day 1, CPI-stabilized emulsions showed smaller particle sizes compared to SPI-stabilized emulsions. For instance, at 3 % protein concentration, CPI-lutein emulsions exhibited an initial particle size of 160.6 ± 3.4 nm, while SPI-lutein emulsions at the same concentration measured 244.3 ± 3.8 nm. This finding is consistent with previous studies that demonstrated improved emulsion stability with CPI or modified SPI due to their strong interfacial activity and protein rearrangement at the interface (M. Zhang et al., 2023).This suggests that CPI forms a more compact and efficient stabilizing layer at the oil-water interface, leading to smaller droplets. Over time, all emulsions showed an increase in particle size, likely due to coalescence and Ostwald ripening. However, the rate of growth varied significantly. CPI-lutein emulsions at 3 % concentration showed better stability, with particle size increasing from 160.6 ± 3.4 nm on Day 1 to 188.6 ± 3.8 nm on Day 15, a modest change compared to SPI-lutein emulsions at 3 %, which grew from 244.3 ± 3.8 nm to 310.6 ± 4.3 nm over the same period. At 5 % protein concentration, CPI also performed better, with smaller size increments compared to SPI-stabilized systems. The results demonstrate that CPI at moderate concentrations (3 %) offers superior droplet size stability, particularly under prolonged storage, compared to SPI. This suggests CPI provides better stabilization for long-term applications, a behavior also observed in emulsions stabilized by protein-modified systems (Zhang et al., 2022). In addition, for The PDI values, which indicate the uniformity of droplet size distribution, also varied based on surfactant type and concentration. At Day 1, CPI-lutein emulsions had lower PDI values compared to SPI-lutein emulsions, reflecting more uniform droplet sizes. For example, CPI at 3 % concentration showed a PDI of 0.268 ± 0.02, while SPI at the same concentration exhibited a PDI of 0.387 ± 0.02. As storage progressed, PDI values increased across all samples, indicating a gradual destabilization of the emulsion. CPI emulsions were steadier and more reliable in terms of PDI values during storage, especially at the 3 % concentration. The values for 3 % with a rise from 0.268 ± 0.02 on Day 1 to 0.294 ± 0.02 on Day 15, and for SPI at the same 3 %, the increase in PDI was more evident from day 1 (from 0.387 ± 0.02 to 0.415 ± 0.03). Thus, STDs reveal that CPI provides more really good behavior at that time when the level of concentration is moderate in comparison to a much worse product - SPI. It is the inclusion of the stronger protein/lipid layers as a result of the protein storing that lowered the PDI in CPI systems and increased uniformity and droplet collision frequency. These are indeed in line with the findings that were obtained from protein-stabilized emulsions in similar studies (X. Cheng et al., 2024). Furthermore, zeta potential values of the phosphate ions give us an idea of the electrostatic stability of the nanoemulsions and the high absolute values are the indicators of good stability against the aggregation. Zeta potential of −18.54 ± 2.54 mV for CPI-stabilized emulsions was significantly higher than the value of −20.31 ± 4.17 mV received for -SPI-stabilized emulsions, meaning droplets interact strongly and in turn no aggregation. In turn, for making CPI solution at the same condition of 3 %, zeta potential remained −30.5 ± 4.1 mV, yet for SPI solution there was a slight decrease, 26.2 ± 3.1 mV. The zeta potencial of the CPI was stable due to the fact that the values decreased slightly to −27.5 ± 3.3 mV on Day 15 in comparison to the initial value. In contrast, SPI-lutein emulsions at 3 % saw a more pronounced decline in zeta potential, dropping from −25.5 ± 2.5 mV on Day 1 to −26.9 ± 3.1 mV on Day 15. At 5 % protein concentration, CPI again outperformed SPI. CPI-lutein emulsions had a zeta potential of −33.5 ± 3.6 mV at Day 1, which only slightly decreased to −30.8 ± 3.8 mV by Day 15. SPI at 5 % concentration showed lower initial values (−31.2 ± 3.8 mV) and a similar decline over time. These results indicate that CPI provides better electrostatic stability for lutein-loaded emulsions, particularly at higher concentrations. This behavior aligns with prior findings where protein modification enhanced emulsion stability by promoting stronger interfacial interactions (D. Zhao et al., 2023). For the addition of lutein influenced the stability of the emulsions. Blank emulsions stabilized by CPI and SPI showed larger particle sizes and lower zeta potential values compared to lutein-loaded systems. For instance, CPI at 3 % without lutein exhibited an initial particle size of 180.1 ± 3.2 nm and a zeta potential of −18.3 ± 2.3 mV, compared to 160.6 ± 3.4 nm and − 27.5 ± 3.3 mV in lutein-loaded systems. This suggests that lutein may contribute to enhanced emulsion stability by forming additional interactions with the protein stabilizers. Overall, CPI-stabilized nanoemulsions exhibited superior long-term stability compared to SPI-stabilized systems. At 3 % concentration, CPI demonstrated the best performance in maintaining particle size, PDI, and zeta potential over 15 days of storage. SPI-stabilized emulsions showed more pronounced changes in these parameters, particularly at lower concentrations (1 %), indicating poorer stability. The results indicate that CPI is a more effective emulsifier for lutein-loaded nanoemulsions, particularly in systems requiring long-term stability. The enhanced stability observed in CPI-stabilized emulsions may be attributed to the stronger interfacial films formed by CPI proteins and their superior surface activity.

**Table 1:** Effect of surfactant type on particle size, scattering index and zeta potential of nanoemulsions and stability over time.

Samples	Particle size (nm) Day			PDI Day			Zeta potential (mV) Day		
	1	7	15	1	7	15	1	7	15
Chickpea 1 %-lutein	231.9± 3.8^e^	299.7± 3.8^c^	410.3± 5.1^d^	0.516± 0.08^b^	0.530± 0.04^b^	0.650± 0.05^a^	−14.8 ±2.5^c^	−15.1 ±2.4^c^	−16.7 ±1.6^c^
Chickpea 3%-lutein	160.6± 3.4^f^	168.3± 3.1^e^	188.6± 3.8^f^	0.268± 0.02^e^	0.276± 0.02^e^	0.294± 0.02^f^	−27.5 ±3.3^b^	−25.3 ±2.5^b^	−26.3 ±2.6^b^
Chickpea 5 %-lutein	253.4± 3.6^c^	405.3± 3.4^a^	434.7± 5.7^c^	0.482± 0.08^c^	0.515± 0.04^c^	0.540 ±0.05^d^	−33.5 ±3.6^a^	−31.3 ±3.8^a^	−30.8 ±3.8^a^
Soybean 1 %-lutein	333.5± 5.2^a^	387.3± 3.8^b^	489.5± 6.3^a^	0.554± 0.07^a^	0.568± 0.03^a^	0.656± 0.04^a^	−11.8 ±1.3^c^	−12.1 ±1.5^c^	−10.9 ±1.3^d^
Soybean 3%-lutein	244.3± 3.8^d^	268.2± 3.3^d^	310.6± 4.3^e^	0.387± 0.02^d^	0.391± 0.02^d^	0.415± 0.03^e^	−25.5 ±2.5^b^	−26.3 ±2.6^b^	−26.9 ±3.1^b^
Soybean 5 %-lutein	343.7± 3.6^b^	398.3± 4.2^a^	498.2± 5.3^a^	0.492± 0.04^c^	0.510± 0.04^c^	0.563± 0.02^c^	−31.2 ±3.8^a^	−29.8 ±3.2^a^	−30.9 ±3.6^a^
Chickpea 3%-blank	180.1± 3.2	290.3± 3.2	410.4± 5.3	0.335± 0.03	0.360± 0.03	0.416± 0.02	−18.3 ±2.3	−17.6 ±2.5	−19.8 ±2.1
Soybean 3%-blank	282.9± 4.1	350.5± 5.1	489.3± 5.4	0.378± 0.02	0.410± 0.02	0.465± 0.02	−15.1 ±1.8	−13.3 ±1.8	−14.8 ±1.6

Different letters indicate significant differences between different concentrations (P < 0.05).

### 3.3 Fourier transform infrared spectroscopy (FTIR)

**Fig. 2:**
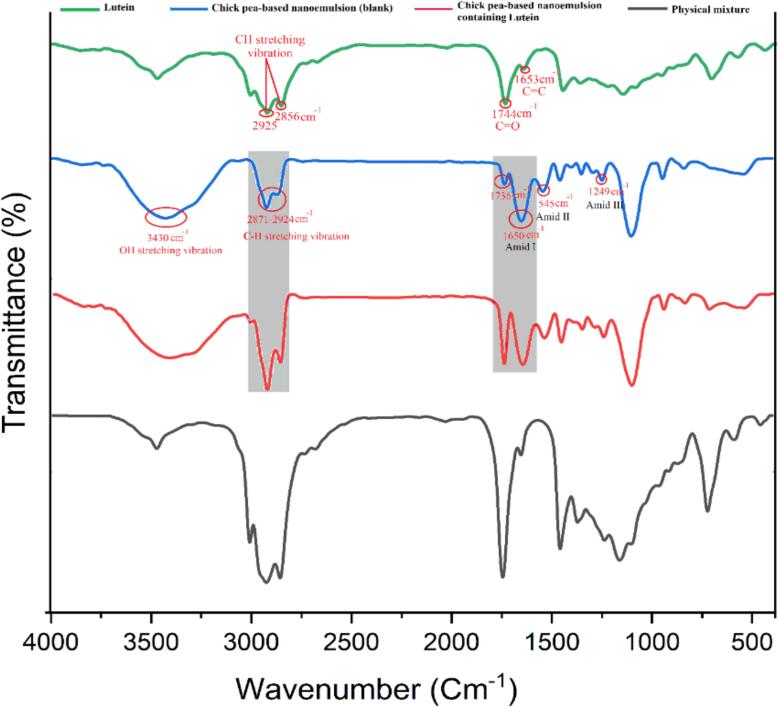
The spectroscopic spectra of pure lutein (green), blank nanoemulsion, which has been stabilized with CPI (blue), CPI-stabilized nanoemulsion containing lutein (red), physical mix of components (gray). (For interpretation of the references to colour in this figure legend, the reader is referred to the web version of this article.)

**Fig. 3:**
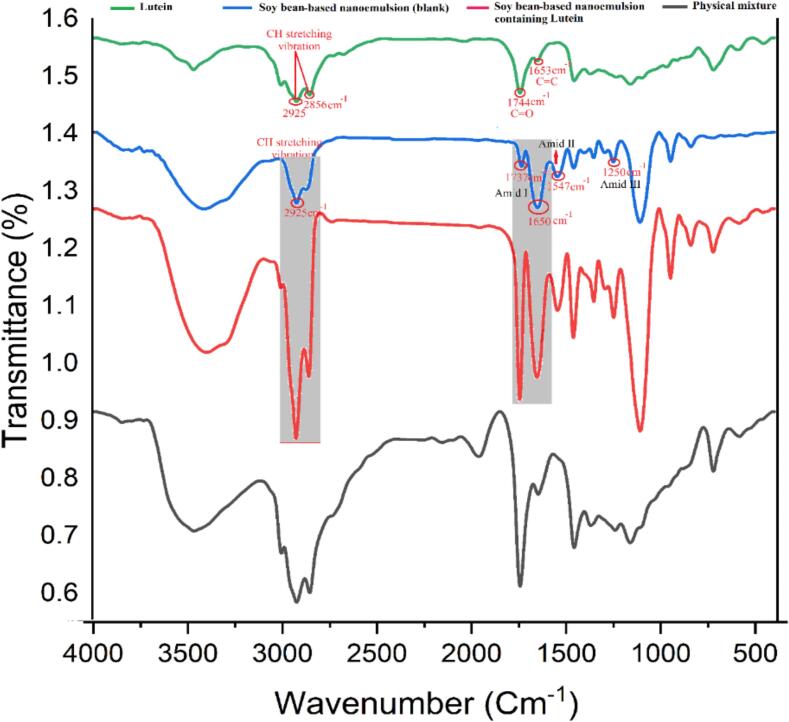
Spectroscopic spectra of pure lutein (green), blank nanoemulsion stabilized with SPI (blue), nanoemulsion stabilized with SPI containing lutein (red), physical mixture of components (gray). (For interpretation of the references to colour in this figure legend, the reader is referred to the web version of this article.)

Fourier Transform Infrared Spectroscopy (FTIR) was employed to investigate the interaction of lutein with (CPI) and (SPI) in nanoemulsions. The results revealed significant structural and chemical bonding changes upon lutein encapsulation, highlighting the role of CPI and SPI in stabilizing lutein in the nanoemulsion systems. In CPI-based nanoemulsions, (Fig. 2) showed distinct absorption peaks indicating strong interactions between lutein and CPI. In the lutein-loaded CPI nanoemulsion, peaks corresponding to C=O stretching at 1744 cm^−1^ and C=C stretching vibrations at 1653 cm^−1^, indicating strong hydrophobic and hydrogen bonding interactions between lutein and CPI. Additionally, a prominent O—H stretching vibration at 3430 cm^−1^ was observed, signifying enhanced hydrogen bonding, which is known to contribute to the stability of lutein in emulsified systems. These findings are supported by studies demonstrating that protein-lutein interactions can lead to structural modifications that improve bioactive retention and stability in functional food systems (Wu et al., 2022). For SPI-based nanoemulsions (Fig. 3), similar C=O and C=C peaks were observed, confirming lutein encapsulation within the SPI matrix. However, the O—H stretching vibration was less pronounced compared to CPI systems, suggesting relatively weaker hydrogen bonding interactions. This aligns with findings indicating that SPI primarily stabilizes lutein through hydrophobic interactions rather than hydrogen bonding, which may slightly reduce its efficacy in maintaining long-term stability (Wu et al., 2022). Both CPI and SPI were found to alter their secondary structures during encapsulation, as evident from changes in amide I and amide II band intensities, further suggesting that lutein integration enhances the functional and structural properties of the proteins. FTIR analysis demonstrated that CPI forms stronger hydrogen bonds with lutein, resulting in greater structural integrity and enhanced stability over time compared to SPI-based nanoemulsions. These results are similar to the studies that demonstrate the protein-lutein interactions might be more effective in the encapsulation efficiency, photostability, and bioavailability of lutein in emulsified systems (Y. Cheng et al., 2024; Yang et al., 2022). In summary, the findings show that the type of protein is the critical factor that determines the physicochemical stability of lutein in nanoemulsions and that the CPI is a stronger system for potential functional food and nutraceutical applications. The use of FTIR in this research shows its success in detail the molecular interactions and structural changes of stable bioactive delivery systems.

### 3.4 Investigating the antioxidant properties of nanoemulsions

The antioxidant properties of (CPI) and (SPI)-based nanoemulsions loaded with lutein were evaluated using DPPH and ABTS radical scavenging assays over a 15-day storage period. The results shown in the Table 2 demonstrate that protein type, concentration, and storage time significantly affect the antioxidant efficiency of the emulsions. Nanoemulsions stabilized with CPI showed consistently higher DPPH and ABTS radical scavenging activities compared to those stabilized with SPI. At 3 % protein concentration, CPI-lutein nanoemulsions displayed superior antioxidant performance, with initial DPPH scavenging activity of 88.1 ± 1.01 % on Day 1, retaining 82.6 ± 3.8 % by Day 15. Just like the ABTS scavenging action was 80.3 ± 1.02 % at the beginning, the value was a bit down to the 71.9 ± 0.92 % after 15 days. Therefore, it was concluded that CPI-based systems maintain the lutein antioxidant properties by better encapsulation of lutein, which is probably because of stronger hydrogen bonds as well as interfacial stabilization effects that are highlighted in a number of recently conducted studies (Liu et al., 2021). Conversely, the nanoemulsions based on SPI at a protein concentration of 3 % were also found to have good antioxidants, with DPPH scavenging activity reaching 80.1 ± 1.4 % on Day 1 and then decreasing to 73.4 ± 1.3 % on Day 15. The ABTS scavenging assay showed a similar behavior, decreasing by 66.8 ± 0.81 % on Day 15 from the initial value of 74.5 ± 1.02 %; however, all these parameters were lower than the CPI-based test emulsions. Thankful for the researchers who explored the topic, it is well known that while SPI creates hydrophobic interactions with lutein, however it has a lower hydrogen bonding capacity compared to CPI, which may cause it uneasy to stabilize lutein and retain antioxidant efficiency (Yang et al., 2022). For lower protein concentrations (1 %), both CPI and SPI nanoemulsions demonstrated a marked decline in antioxidant capacity over time, indicating the importance of higher protein concentrations for maintaining stability and effectiveness. CPI at 1 % showed 54.1 ± 1.1 % DPPH pharmacy activity on Day 15, while SPI at the same concentration showed only 48.4 ± 1.3 %. This pattern reveals even better CPI's encapsulation and stabilizing effectiveness, as found in protein-based emulsifiers antioxidative properties (Akbarbaglu et al., 2023). Blank nanoemulsions without lutein lead to a marked decrease in the antioxidant activity of the emulsions, which emphasizes the crucial role of lutein as the real antioxidant agent in these systems. For example, CPI blanks that are 3 % protein concentration only had 19.3 % ± 0.98 % DPPH scavenging activity on Day 15, while 82.6 % ± 3.8 % was accomplished in lutein-loaded emulsions. Ultimately, the CPI-based nanoemulsions outperformed those stabilized using the SPI method in terms of antioxidant retention among all concentrations and time points. These discoveries are in line with the present research demonstrating that CPI-derived nanoemulsions are superior in the stability and bioavailability of bioactive compounds such as lutein, thus making them be the best choice for functional food and nutraceutical applications (S. Wang et al., 2023).

**Table 2:** DPPH and ABTS radical scavenging activity (%) of chickpea and soybean protein isolate-based nanoemulsions loaded with lutein at different concentrations (1 %, 3 %, and 5 %) over a 15-day storage period were studied.

Samples	%DPPH scavenging Day			%ABTS scavenging Day		
	1	7	15	1	7	15
Chickpea 1 %-lutein	68.2± 1.1^c^	60.1± 1.8^d^	54.1± 1.1^d^	58.4± 0.91^d^	49.3± 1.01^d^	41.4± 1.08^e^
Chickpea 3 %-lutein	88.1± 1/01^a^	86.2± 1.1^a^	82.6± 3.8^a^	80.3± 1.02^a^	77.6± 1.02^a^	71.9± 0.92^a^
Chickpea 5 %-lutein	83.2± 1.5^b^	80.2± 1.4^b^	72.8± 1.7^b^	75.3± 0.91^b^	68.9± 1.01^b^	61.9± 1.01^c^
Soybean 1 %-lutein	68.2± 1.2^c^	59.5± 1.8^d^	48.4± 1.3^e^	56.7± 1.05^d^	46.9± 1.08^d^	38.6± 1.04^e^
Soybean 3%-lutein	80.1± 1.4^b^	78.2± 1.3^b^	73.4± 1.3^b^	74.5± 1.02^b^	70.9± 1.04^b^	66.8 ± 0.81^b^
Soybean 5 %-lutein	76.7± 1.5^c^	71.1± 1.2^c^	64.2± 1.2^c^	69.7± 1.04^c^	62.6± 1.04^c^	56.9± 1.02^d^
Chickpea 3%-blank	27.89± 1.2	25.8± 1.2	19.3± 0.98	22.4± 0.83	18.9± 1.03	15.1± 1.01
Soybean 3%-blank	26.5± 1.5	23.5± 1.1	17.8± 1.4	20.5± 0.92	16.9± 1.02	13.4± 0.92

Data express the mean ± SD, where the various letters indicate statistically significant differences between the means of the samples (p < 0.05). The average measurements in the table are presented as the mean value ± standard deviation (*n* = 3).

### 3.5 Morphology of nanoemulsions

The protein (CPI) and the (SPI)-stabilized lutein-based nanoemulsions were observed and analyzed to clarify the structural characteristics using both Transmission Electron Microscopy (TEM) and Scanning Electron Microscopy (SEM). Various TEM images showed distinct round shapes of nanoparticles differing by size and at the same time demonstrating efficient encapsulation and stable Lutein throughout the CPI and SPI matrices. Nanoemulsions were stabilized by CPI that showed slightly smaller particles and denser structures compared to SPI, matching analogous studies that attribute better lutein encapsulation efficiency and stability to stronger protein-lutein interactions (Wu et al., 2022). Additionally, SEM imaging confirmed the observations and showed smooth homogeneous surfaces for CPI-stabilized systems, which meant that the interfacial films formed by the chemical were stable. The SPI-based emulsions exhibited some effect but they too were bigger in size and not very compact in morphological respect, which could adversely affect lutein's stability and release properties. Similarly, these results are in concordance with the earlier findings that soft and surfactant properties of CPI entitle it as a better candidate in the stable nanoemulsion formation (Xu et al., 2021).

TEM and SEM were used together, and the results were crucial for the in-depth visualization of the lutein-carrying emulsions, showing that the antioxidant Institute for Food Safety and Health is an ideal stabilizer and nanoemulsion for enhanced the structural integrity and many potential applications in the functional food and nutraceutical systems (Y. Wang, X. Zhang, et al., 2023).

### 3.6 Analyzing the efficiency of encapsulation and chemical stability of nanoemulsions

**Fig. 4:**
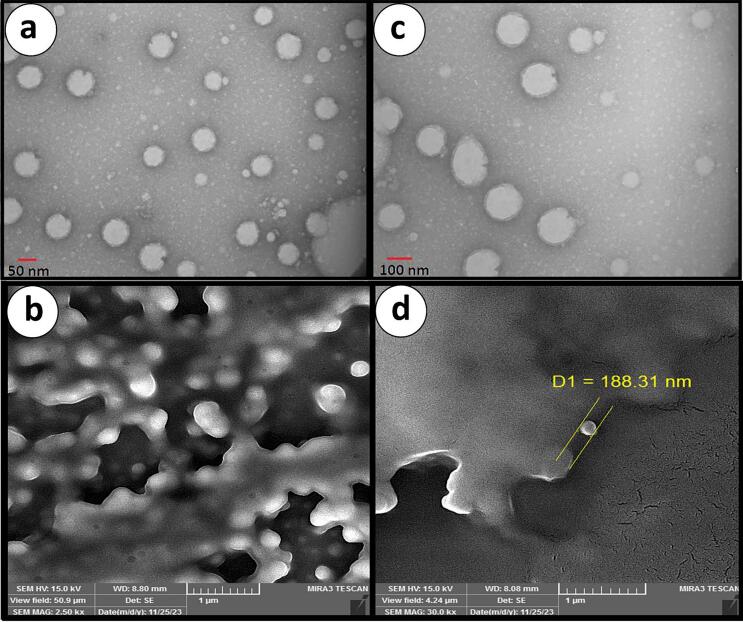
SEM and TEM CPI-stabilized emulsion with lutein in (a,b), SPI-stabilized emulsion with lutein in (c,d).

The encapsulation efficiency and chemical stability of lutein-loaded nanoemulsions stabilized by (CPI) (SPI) were evaluated over a 15-day storage period. It was found, as it is shown in Fig. 4, that CPI-stabilized nanoemulsions were the most effective over time in maintaining the chemical stability of lutein. At a 3 % protein concentration, CPI nanoemulsions kept the chemical stability at the level above 85 % on the 7th and at 80 % on the 15th day, while SPI-stabilized systems with the same protein concentration got worse, they retained only 70 % on the 15th day. These results emphasize the high efficiency of CPI in encapsulation, which is justified by the formation of robust interfacial films, with huger abilities to defend lutein against oxidative and environmental degradation (Leng et al., 2022). A study demonstrated that nanoemulsions using chickpea protein isolate and stevioside (CPI-STE) achieved high encapsulation efficiency (87.56 %) and increased stability of lutein in gastrointestinal phases. Hydrogen bonding was identified as the key mechanism for CPI-lutein interaction (Xu et al., 2021). Protein concentration showed the key in characteristic to determine the chemical stability of the nanoemulsions. Among the three protein concentration levels, the CPI-stabilized emulsions with 3 % protein were determined to be the most efficient, maintaining the chemical stability better than both 1 % and 5 % concentrations. At the lowest level, low protein content failed to form strong interfacial stabilization, and the consequence was that lutein degraded more quickly. While at the highest level, the excessive protein content might have caused aggregation, so the lutein encapsulation efficiency was reduced. These results are in line with other studies demonstrating that the ideal protein concentration is the one that allows the formation of a dense layer in the interfacial region and hence limits the oxidative degradation and droplet instability (Nunes, Hashemi, Gregersen, et al., 2023). The comparison of CPI and SPI surprisingly favored CPI for the mentioned purpose of being an emulsifier. The results of nanoemulsions stabilized with CPI revealed stronger protein-lutein interactions and less stable decay over the storage period. The essential feature of CPI to establish hydrogen bonding, as well as the formation of dense interfacial networks, protected lutein effectively. SPI-stabilized systems have displayed a certain affectivity as well, but it is being weakened due to the hydrophobic interactions that are the main factor, so it is not enough to maintain strong protect against oxidative degradation over time (Cheng et al., 2025). High-pressure homogenization and trypsin hydrolysis of SPI enhanced lutein encapsulation efficiency and photostability. For example, enzymatic treatment increased encapsulation efficiency by up to 18.59 % compared to untreated SPI, highlighting improved structural compatibility and antioxidant properties(Y. Cheng et al., 2024). Lutein presence significantly improved emulsion stability as compared with respective blanks. The stability of the nanoemulsions without lutein to their CPI and SPI blends was only marginally lower. The vast reduction in lutein showed that this carotenoid induces an important structural change to allow the amount of the emulsions to change. This, in turn, would imply the effectiveness of the emulsion stabilizer, most likely, lutein (Ge et al., 2024). Over a 15-day period, the CPI-based nanoemulsion appeared to exhibit its own stability without chemical degradation and it was materially different from the SPI equivalent that was less stable. For instance, the 3 % CPI nanoemulsion stayed at 80 % concentration of its initial state. On the other hand, the SPI types were around 70 %, the nanoemulsions retained their stability. Besides, the CPI ingredients enhance the stability and functional properties and maintain their specified characteristics even after a long time. High performance and CPI being an effective emulsifier for functional food and nutraceuticals that require long-term stability and bioavailability of bioactives like lutein are the main takeaways (D.-b. Zhao et al., 2023). In addition SPI combined with genistein and modified under high-pressure conditions yielded nanoemulsions with encapsulation efficiency up to 93.88 % and good storage stability over 20 days, suggesting the importance of SPI modifications to achieve better performance (Li et al., 2023). According to the present study, the right selection of emulsifier and concentration of protein for lutein-loaded nanoemulsions can determine the final result if the study shows that CPI is higher in terms of encapsulation efficiency and chemical stability than SPI, especially at the moderate concentration. These results point out the possibility of the use of CPI in the production of stable and bioavailable lutein delivery systems in functional foods and nutraceuticals as a bioactive agent with positive controlled behavior to enhance the development of these systems. Although this study evaluated stability over a 15-day period, future work should extend the storage time to several months to assess long-term performance under real-world conditions, especially for shelf-stable functional food applications.

### 3.7 Creaming index (CI) of nanoemulsions

Physical stability of lutein-loaded nanoemulsions was determined by the creaming index (CI) evaluation out under the influence of (CPI) and (SPI) over 15 days of storage. The results, as summarized in the provided Table 3, demonstrated a clear influence of emulsifier type, protein concentration, and storage time on the stability of the nanoemulsions. At Day 1, all samples exhibited a creaming index of 0 %, which means that initially, the nanoemulsions were not agglomerated and were stable. The oil-water interface contains the interfacial monolayer made by CPI and SPI, therefore, it is the “growing” factor in this case, which prevents droplet coalesced. However, as storage time increased, notable differences in creaming index were observed among the samples. Nanoemulsions stabilized with CPI demonstrated better long-term stability compared to those stabilized with SPI. At 3 % protein concentration, CPI-lutein nanoemulsions maintained a very low creaming index (3.12 ± 0.39 %) at Day 15, highlighting their superior ability to prevent phase separation. The lutein in nanoemulsions that had been treated them with SPI at the same concentration in this period showed a somewhat higher creaming index (4.82 ± 0.39 %) on Day 15, which means a slightly reduced stability. The result of this study is in line with the earlier study that had shown CPI to form a more compact interfacial layer because of the stronger protein-protein interactions and the hydrogen-bonding (Liu et al., 2024). Both CPI and SPI demonstrated significantly greater creaming indices by Day 15 in 1 % protein formulations. Where CPI-lutein nanoemulsions peaked at 30.72 ± 1.48 %, SPI-lutein nanoemulsions reached 31.72 ± 2.22 %. These increased measures signify that lower protein concentrations are related to the decrease in stability because there is not enough emulsifier to ensure a good, continuous and protective interfacial layer. At the higher protein concentration of 5 %, CPI stabilized nanoemulsions showed better stability than SPI. On the fifteenth day, the CPI-lutein nanoemulsions had a lower creaming index of 25.32 ± 1.78 %, which was a bit higher when compared to 29.42 ± 2.12 % obtained for SPI-lutein nanoemulsions. These findings indicate that higher protein concentrations are essential to provide better stabilization of emulsions by the CPI method, which is likely due to higher surface activity and its ability to produce a denser network formation around the droplets (Hei et al., 2024). Empty lube emulsions (without lutein) were up to 10 times faster than the lutein-loaded emulsions in showing emulsion instability. For example, CPI's lube emulsions (at 3 % protein) with a cream index of 31.32 ± 1.82 % showed the highest creaming index after 15 days, whereas the CPI-lutein emulsions demonstrated the lowest creaming index of 3.12 ± 0.39 % at the same protein concentration and time. At the same time, SPI-blank emulsions at 3 % protein concentration had creaming index values of 35.42 ± 2.28 and 4.82 ± 0.39 for the SPI-lutein emulsions at the same date. The findings of this study propose that the lutein is a contributor to the stability of the nanoemulsion by enhancing the creation of additional hydrophobic and hydrogen bonding within the media (Yang et al., 2022). To summarize, compared to SPI-stabilized creaming stability at all protein concentrations, the CPI-stabilized nanoparticles stably performed across all protein concentrations. The data suggest that the moderate protein concentration of 3 % is optimal for both CPI and SPI systems in terms of stability.

**Table 3:** CI values in emulsions formulated with various CPI and SPI concentrations kept at the ambient temperature of 25 °C and stored for the duration of 15 days.

Samples	Creaming index (%) Day		
	1	7	15
Chickpea 1 %-lutein	0.00 ± 0.00^a^	5.48 ± 0.78^b^	30.72 ± 1.48^a^
Chickpea 3 %-lutein	0.00 ± 0.00^a^	0.00 ± 0.00^c^	3.12 ± 0.39^c^
Chickpea 5 %-lutein	0.00 ± 0.00^a^	5.32 ± 0.88^b^	25.32 ± 1.78^b^
Soybean 1 %-lutein	0.00 ± 0.00^a^	7.42 ± 1.01^a^	31.72 ± 2.22^a^
Soybean 3 %-lutein	0.00 ± 0.00^a^	0.00 ± 0.00^c^	4.82 ± 0.39^c^
Soybean 5 %-lutein	0.00 ± 0.00^a^	5.32 ± 0.88^b^	29.42 ± 2.12^a^
Chickpea 3 %-blank	0.00 ± 0.00^a^	8.32 ± 0.39^a^	31.32 ± 1.82^a^
Soybean 3 %-blank	0.00 ± 0.00^a^	7.52 ± 0.47^a^	35.42 ± 2.28^a^

Different letters indicate significant differences between different concentrations (*P* < 0.05).

### 3.8 Stable rheological properties

**Fig. 5:**
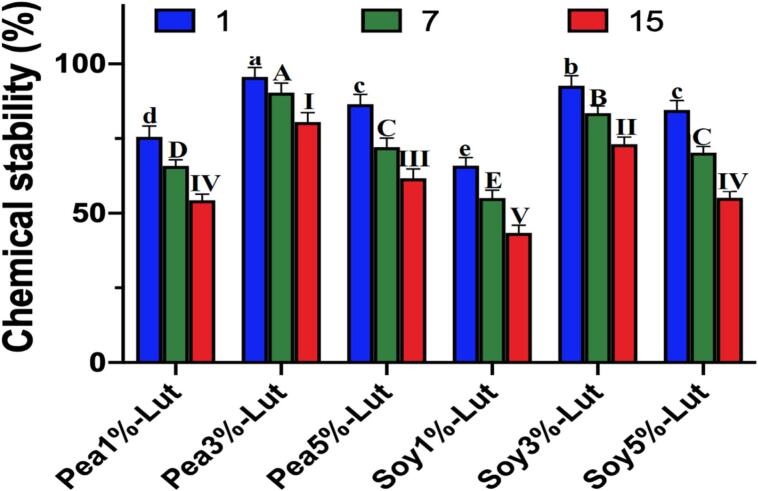
Emulsions of lutein with different levels of CPI and SPI SBSM during 1, 7 and 15 days were tested for chemical stability.

The sheer viscosity is one of the essential parameters describing the flow-specific properties of emulsions, that is to say, directly affecting the appearance, consistency, stability of the system (Grossi Bovi Karatay et al., 2022). The impact of lutein-loaded nanoemulsions stabilized by CPI and SPI to the apparent viscosity of different protein concentrations (1 %, 3 %, and 5 %) at varying shear rates is presented in Fig. 5. At a shear rate of 0.01 s^−1^, the apparent viscosity of CPI-stabilized nanoemulsions was found to be greater with the increase of protein concentration, as its values were 3.11 mPa·s (1 %), 4.15 mPa·s (3 %), and 7.56 mPa·s (5 %) respectively. Comparatively, SPI-stabilized nanoemulsions exhibited lower apparent viscosity values of 2.31 mPa·s (1 %), 3.34 mPa·s (3 %), and 6.9 mPa·s (5 %) under the same conditions. Differences between CPI and SPI indicate that CPI creates a more tenacious interfacial network than SPI, thus the acceleration of viscosity and stability is increased. By Stoke's formula, the pace at which solid particles move is inversely related to their viscosity. Polymers such as hydrocolloids (CPI and SPI) came into existence as the flow of the continuous phase was increased, this was followed by particle movement blocking, hence, self-assembly of the particles. As a result, the stability of the system against coalescence and creaming was achieved. The results of the creaming index and particle size tests were consistent, with 3 % minced CPI based nanoemulsions exhibiting minimal particle aggregation and the highest stability. These results mean that if you increase the specific and steady census of CPI to 3 %, and it in turn, leads to more emulsification, then it will mainly jibe with the data from the experiment. Consequently, as protein concentration was rising, there was an increase in intramolecular interaction that was reducing the molecular mobility hence the results of higher viscosity values and improved stabilization of the emulsions (Lv et al., 2023). This led to insufficient coverage of the biopolymer around the oil droplets causing droplet aggregation and a modest increase in viscosity. Growth in protein concentration (5 %) was enough such that the protein was around the droplets, thus an increase in viscosity through repulsive forces and interfacial coverage was achieved (Dammak & Sobral, 2018). It is the best way to ensure the proper protein concentration is maintained which makes the system stable and can last longer. On the other hand, from 0.1 s^−1^ to 100 s^−1^, it can be seen that the nanoparticles all exhibit shear-thinning (pseudoplastic), during which the apparent viscosity decreases due to the destruction of the structure and the breakdown of the chain during the shear process (Zhao et al., 2022). It is also evident that the same behavior profile is observed in emulsions with different oil-protein combinations such as soy and pea protein isolates, which also correlates with the results of this experiment (Ćirin et al., 2023; Wang et al., 2020). Shear-thinning (pseudoplastic) behavior enhances the mixing and pumping process by creating stable and processable structures and thus is especially beneficial in food and nutraceutical applications. Lutein which is a polar molecule of a xanthophyll pigment is localized on the surface of the amphiphilic shell of emulsion droplets where it can interact with proteins such as CPI and SPI and, thus, it can alter physical and rheological parameters of the nanoemulsions. It is assumed that these compounds will provide much better results in highly stable, lutein loading emulsions than SPI will give. It is a well-documented fact that lutein, being a polar carotenoid, causes strong interactions to emerge in the emulsion and thus increased viscosity (Li et al., 2024). In summary, CPI-stabilized nanoemulsions exhibit higher apparent viscosity and better stability than SPI-stabilized ones particularly at 3 % concentration. The findings further prove that CPI has a better emulsifying property compared to SPI and it could be used in producing functional food and nutraceutical applications.

### 3.9 Thixotropic test

**Fig. 6:**
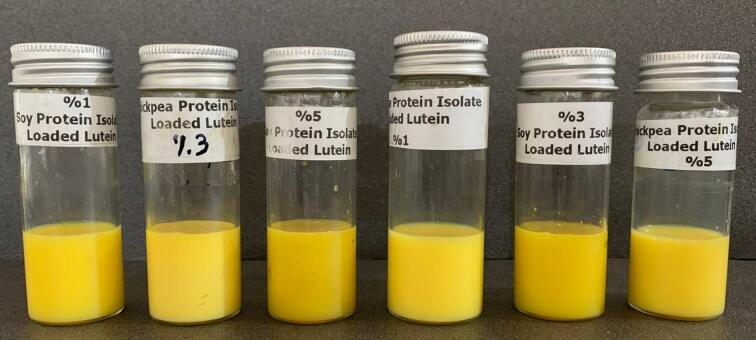
On the first day of storage, the stability of emulsion samples creamed at different concentrations of CPI and SPI which contained lutein.

**Fig. 7:**
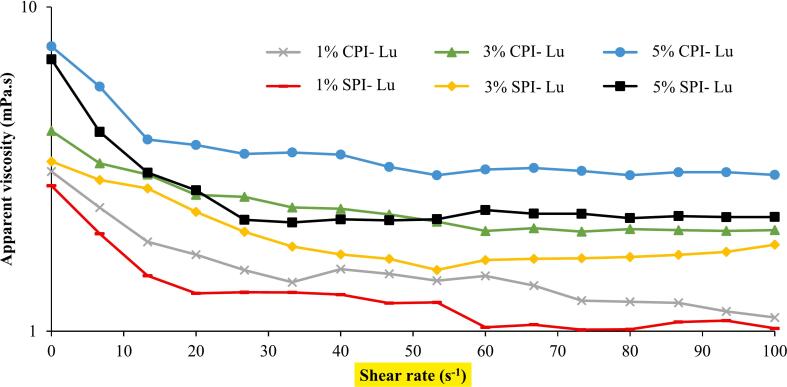
The relationship between apparent viscosity and shear rate of nanoemulsions prepared from different concentrations of CPI and SPI containing lutein (Lu) (25 °C).

**Fig. 8:**
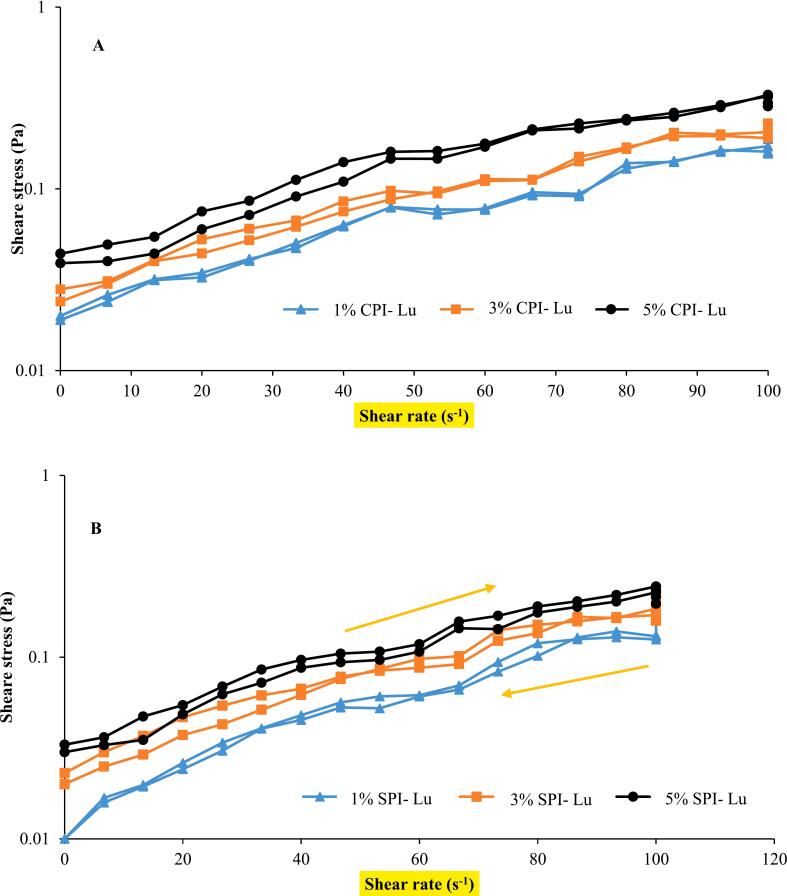
The hysteresis loop of the flow curve of nanoemulsions that were made from different concentrations of (a)CPI and (b) SPI containing lutein (Lu) at 25 °C.

Thixotropic describes the time-dependent shear thinning behavior of a fluid, where the apparent viscosity decreases with continuous shear at a constant rate (Arab, Ghanbarzadeh, Karimi, Ebrahimi, & Hosseini, 2023). In thixotropic fluids, the shear stress-shear velocity diagram forms a hysteresis loop due to the non-coincidence of upward and downward curves. The area of the hysteresis loop quantifies the degree of thixotropy, with larger areas indicating stronger thixotropic properties and greater time dependency (Karimi, Ghanbarzadeh, Roufegarinejad, & Falcone, 2022). Thixotropic systems generally exhibit greater stability due to their ability to form three-dimensional networks, which prevent rapid structural breakdown and enhance stability over time. The results presented in Fig. 6 and Table 4 demonstrate that CPI- and SPI-stabilized lutein-loaded nanoemulsions exhibit varying degrees of thixotropic behavior based on protein concentration. At 1 % and 3 % concentrations, the nanoemulsions showed weak thixotropic properties, with minimal hysteresis loop formation. These results suggest that the structural integrity of the protein-lipid interface is not sufficient to maintain a strong three-dimensional network at lower protein concentrations. However, when the protein concentration increased to 5 %, both CPI and SPI samples exhibited clear hysteresis loops, indicating pronounced thixotropic behavior and a stronger polymer network. The hysteresis loop area for 5 % CPI-stabilized nanoemulsions was 0.68 Pa.s^−1^, compared to 0.43 Pa.s^−1^ for SPI-stabilized systems, highlighting CPI's superior thixotropic properties and network strength. The stronger thixotropic behavior observed in 5 % CPI-stabilized nanoemulsions ‘conforms to the information that’ CPI forms more cohesive interfacial layers due to its superior hydrogen bonding and surface activity. This leads to a more robust three-dimensional network that resists structural breakdown under shear and quickly reforms upon shear removal. Interestingly, SPI nanoemulsions at 5 % protein concentration exhibited weaker thixotropic behavior, which may be attributed to its reliance on hydrophobic interactions rather than the combination of hydrophobic and hydrogen bonding seen with CPI (Domian & Szczepaniak, 2020). Thixotropic behavior is also affected by droplet aggregation and system uniformity. Nano systems with 3 % protein concentration were demonstrated to be of the most stable nature with most of the proteins covering the oil droplets uniformly and the lowest aggregation. Conversely, at 1 %, insufficient protein coverage led to unstable droplets and weak thixotropic behavior. With 5 % the higher protein concentration led to the formation of the three-dimensional network and as a result the stability and the thixotropic properties of the emulsions were both improved (Krstonošić et al., 2024). Previous studies confirm these findings. For instance, the study by (Lu et al., 2023) showed that only pea protein isolate emulsions (1 %) containing curcumin had a brief period of broken structures, but the broken structures changed to a similar solid state. Moreover, (Giorgio, Salgado, & Mauri, 2022) witnessed thixotropic behavior in 5 % soy protein isolate emulsions, the formation of a three-dimensional network being the main factor that allowed the system to be more stable. In summary, the thixotropic property of lutein-loaded nanoemulsions largely relies on protein type and concentration. The CPI-stabilized nanoemulsions showed robust thixotropic properties compared to the SPI-stabilized systems, especially at higher protein concentrations since CPI can develop a very strong three-dimensional structure. These results highlight the potential of CPI as a better emulsifier for lutein-fortified stable and functional nanoemulsions. (See Fig. 7, Fig. 8.)

**Table 4:** Values of apparent viscosity (at shear rate 0.1 s^−1^) and hysteresis loop area of nanoemulsions.

Nano emulsion	Apparent viscosity (Pa.s)	Hysteresis loop area (Pa.s^−1^)
Chickpea 1 %-lutein	3.11	0.001
Chickpea 3 %-lutein	4.15	0.34
Chickpea 5 %-lutein	7.56	0.68
Soybean 1 %-lutein	2.32	0.006
Soybean 3%-lutein	3.34	0.11
Soybean 5 %-lutein	6.10	0.43

## 4 Conclusion

This research report is about comparing how (CPI) is comparatively a better emulsifier (SPI) as far as the stabilization of lutein-loaded nanoemulsions is concerned. CPI presented characteristics suggesting that it could disperse the large number of oil droplets evenly, especially at as little as 3% efficiency, thus offering the desired droplet size and zeta potential with quite a high rate of separation. The bond energy and the layer's cohesive forces in CPI caused the nanoemulsion to be significantly strengthened and prevent the loss of the antioxidants and the rheological stability by its thixotropic frequency and creaming rate of time. Furthermore, the addition of lutein also plays a significant role in the stable delivery of the nanoemulsions and enhancement of the antioxidant properties, which support the idea that the emulsifier-bioactive interaction is the key to functional and stable delivery systems. Although SPI also performed well, its dependence on hydrophobic interactions was a restriction that rendered it unable to achieve the same stability and overall efficacy as CPI. The results of this study introduce CPI as an eco-friendly alternative and an innovative plant-based emulsifier for use in nanoemulsion compositions and thus, in the development of functional foods and nutraceuticals this study demonstrate potential for practical applications in commercial functional food and nutraceutical products, including fortified beverages (e.g., eye-health drinks), dairy-based systems (e.g., yogurts), and dietary supplements (e.g., soft-gel capsules or gummies). Through developing new and stable bioactive compounds like lutein, that are the base of plant-based and health-promoting products, professionals in the health sector are able to use CPI's emulsifying proficiency and provide sustainable safe delivery systems together with bioavailability to bioactive ingredients. Future research should investigate the interfacial behavior of CPI and SPI in nanoemulsion systems, including protein adsorption kinetics, interfacial rheology, and molecular rearrangement, to more comprehensively understand the stabilization mechanisms at the oil–water interface.

## CRediT authorship contribution statement

**Farhang Hameed Awlqadr:** Writing – original draft, Validation, Methodology, Investigation, Conceptualization. **Babak Ghanbarzadeh:** Writing – review & editing, Resources, Methodology, Conceptualization. **Ammar B. Altemimi:** Writing – review & editing, Validation, Methodology, Data curation. **Khaled Arab:** Writing – original draft, Validation, Methodology, Investigation, Formal analysis. **Saeed Dadashi:** Writing – review & editing, Methodology, Investigation, Formal analysis. **Akram Pezeshki:** Writing – review & editing, Methodology, Formal analysis. **Mohammad Ali Hesarinejad:** Writing – review & editing, Visualization, Software. **Tarek Gamal Abedelmaksoud:** Writing – review & editing, Visualization, Software.

## Declaration of competing interest

The authors declare that they have no known competing financial interests or personal relationships that could have appeared to influence the work reported in this paper.

## Data Availability

Data will be made available on request.
